# Family planning counseling for women living with HIV: a systematic review of the evidence of effectiveness on contraceptive uptake and pregnancy incidence, 1990 to 2011

**DOI:** 10.1186/1471-2458-13-935

**Published:** 2013-10-08

**Authors:** Kevin R O’Reilly, Caitlin E Kennedy, Virginia A Fonner, Michael D Sweat

**Affiliations:** 1 Department of HIV/AIDS, World Health Organization, Geneva, Switzerland; 2 Department of International Health, Johns Hopkins Bloomberg School of Public Health, Baltimore, MD, USA; 3 Department of Psychiatry and Behavioral Sciences, Medical University of South Carolina, Charleston, SC, USA

**Keywords:** HIV, Family planning, Counseling, Systematic review

## Abstract

**Background:**

Family planning is an important public health intervention with numerous potential health benefits for all women. One of those key benefits is the prevention of mother-to-child transmission of HIV, through the prevention of unintended pregnancies among women living with HIV.

**Methods:**

We conducted a systematic review of the effectiveness of family planning counseling interventions for HIV infected women in low- and middle-income countries.

**Results:**

We found nine articles which met the inclusion criteria for this review, all from Africa. Though these studies varied in the specifics of the interventions provided, research designs and measures of outcomes, key features were discernible. Providing concerted information and support for family planning use, coupled with ready access to a wide range of contraceptive methods, seemed most effective in increasing use. Effects on pregnancy overall were difficult to measure, however: no studies assessed the effect on unintended pregnancy.

**Conclusions:**

Though these results are far from definitive, they do highlight the need for strengthened efforts to integrate family planning counseling and access to services into HIV prevention, and for greater consistency of effort over time. Studies which specifically investigate fertility intentions and desires of women living with HIV, contraception use following interventions to increase knowledge, awareness, motivation and access to the means to act on those intentions and unintended pregnancies would be valuable to help clinic personnel, programme planners and policy makers guide the development of the integrated services they offer.

## Background

An estimated 34 million people were living with HIV in 2011 [1]. Of these, nearly three and a half million were children under the age of 15, the great majority of whom lived in sub-Saharan Africa and were likely to have been infected at birth. In 2011, an estimated 330,000 children were newly infected with HIV globally, more than 90% of them through mother-to-child transmission [1]. With the interventions currently recommended by WHO^a^ the risk of mother to child transmission can be reduced to less than 2% in non-breastfeeding settings, and to 5% or less in breastfeeding populations [2].

The United Nations has recently set a goal of reducing the number of new HIV infections among children by 90% and the number of AIDS-related maternal deaths by 50% by 2015 [3]. To prevent mother to child transmission, the World Health Organization (WHO) promotes four key elements: [1] the primary prevention of HIV infection in women of childbearing age, especially young women [2] the prevention of unintended pregnancies in women living with HIV, [3] the prevention of vertical transmission from a woman living with HIV to her infant, and [4] the provision of treatment, care and support to mothers living with HIV and their children and families [4,5]. These efforts have been guiding PMTCT programmes in the last decade. Strengthening attention to this goal at country level has become a key priority.

Global PMTCT efforts have focused primarily on the third element of the PMTCT strategy: the provision of antiretroviral drugs to pregnant women living with HIV to prevent vertical transmission. In its newest guidance, PEPFAR has stated that funds may not be used to purchase family planning commodities but can be used to support integration of family planning services and HIV prevention in PEPFAR-funded PMTCT and HIV care and treatment programmes, using commodities funded by sources other than PEPFAR [6]. The availability of those funds from other sources has also been limited.

Contraception offers a range of health benefits to all women and their families, not just to those living with HIV [7]. Several authors have highlighted the substantial contribution that family planning can make to reducing mother-to-child transmission of HIV by decreasing the frequency of unintended pregnancies among women living with HIV [8,9]. At the global level, policy support for the integration of reproductive health, especially family planning, into HIV/AIDS programmes, especially PMTCT, has been established [10-12]. Essential services for preventing unintended pregnancies among women living with HIV have been defined [13]. In addition, family planning for women living with HIV not only can help prevent mother-to-child HIV transmission, but also can provide additional health and social benefits for women and their children. Coupled with an increasingly strong call for respect of the human rights and reproductive choices of women living with HIV [14], accelerated efforts to reduce new HIV infections in children now call for strengthened integration of reproductive health services, especially family planning services, and HIV/AIDS programmes. Given this increased emphasis on integration at the global and programmatic level, we undertook a systematic review of the evidence of family planning counseling for women living with HIV, specifically the effect of interventions on increasing contraceptive use and decreasing the incidence of unintended pregnancy.

## Methods

This review is part of a series of systematic reviews of HIV behavioral interventions in low- and middle-income countries conducted for the Evidence Project, a collaboration between the World Health Organization, the Medical University of South Carolina, and the Johns Hopkins Bloomberg School of Public Health. Other interventions that have been systematically reviewed through the Evidence Project include voluntary counseling and testing (VCT) [15], mass media [16], psychosocial support [17], treatment as prevention [18], peer education [19], positive prevention [20], condom social marketing [21] and provider-initiated testing and counseling [22]. For this project, we follow established PRISMA reporting guidelines [23] and use standardized methods across reviews. In this review, we sought to answer the question, in low and middle income countries, what is the impact of family planning counseling for women living with HIV on HIV/AIDS-related outcomes?

### Inclusion criteria

Inclusion criteria for articles were as follows:

1) Published in a peer-reviewed journal.

2) Intervention provided family planning counseling (one-on-one counseling, not just health education) to HIV-infected women. Articles included could either: [1] present data from HIV-infected women only, or [2] present data from both HIV-infected and HIV-uninfected women. To be included in the review, articles in the second category had to present pre-intervention / post-intervention or multi-arm data separately for HIV-infected women.

3) Analysis used a pre/post or multi-arm design, comparing individuals who received family planning counseling to those who did not to allow an assessment of post-intervention outcomes of interest.

4) Analysis included at least one HIV-related behavioral, psychological, social, care or biological outcome. For purposes of this review we included pregnancy in women living with HIV as an “HIV-related” biological outcome.

5) Study conducted in a low, lower-middle, or upper-middle income country, according to the World Bank country classification scheme.

No restrictions were placed based on location or settings of the intervention. No language restrictions were used on the search; studies identified in languages other than English were translated for inclusion.

### Study designs

Any pre-post or multi-arm intervention study comparing individuals or groups who received family planning counseling to those who did not was included for review. Family planning counseling was defined as more than information or education about contraception; health education or the provision of factual information without personal counseling was not sufficient for inclusion. Family planning counseling could be linked to or part of VCT activities, or to HIV care and treatment, or could be provided independently. Studies were included if they sampled either individuals or groups who received the intervention versus those in a control or comparison group, or individuals or groups before and after receiving the intervention.

### Search strategy

We searched PubMed, PsycINFO, Sociological Abstracts, CINAHL (Cumulative Index to Nursing and Allied Health Literature), and EMBASE using the date ranges January 1, 1990 to December 31, 2011. In addition, we reviewed the table of contents of four journals: *AIDS, AIDS and Behavior, AIDS Education and Prevention,* and *AIDS Care.* Secondary reference searching was conducted on the reference lists of articles included in the study.

### Search terms

The following search terms were entered into each online database: (“family planning” OR “birth control” OR contraceptive OR contraception OR fertility OR “reproductive counselling” OR “reproductive counseling” OR “reproductive planning” OR “birth spacing”) AND (HIV-infected OR HIV-positive OR “women living with HIV” OR “people living with HIV” OR “HIV seropositive” OR “infected with HIV” OR “living with AIDS” OR “HIV seropositivity”).

### Screening abstracts

Citations identified through the search strategy were initially reviewed for inclusion based on information contained in titles, abstracts, citation information, and key words by a single reviewer. The relevant citations were then independently screened by two reviewers to determine eligibility. Full text articles were obtained for all eligible studies and for those which needed further review to determine eligibility. Background articles that contained relevant information but did not meet the inclusion criteria were also obtained.

### Data extraction and management

Two reviewers extracted data independently using standardized data extraction forms. Differences in data extraction were resolved through consensus and discussion with a senior study team member when required.

Data extraction forms gathered the following information from each included study: location, setting & target group; years (period of study); description of the intervention; study design; sample size; age range, gender; random or non-random allocation of participants; length of follow-up; outcome measures; comparison groups; effect sizes; confidence intervals; significance levels; and limitations identified by authors and reviewers. Study quality (rigor) was assessed based on an 8-item list developed by The Evidence Project for similar reviews. Background studies were assessed using a greatly simplified data extraction form.

### Data analysis

Data were analyzed descriptively; meta-analysis could not be conducted due to the heterogeneity of interventions, populations, measured outcomes, and study designs.

## Results

**Figure 1:**
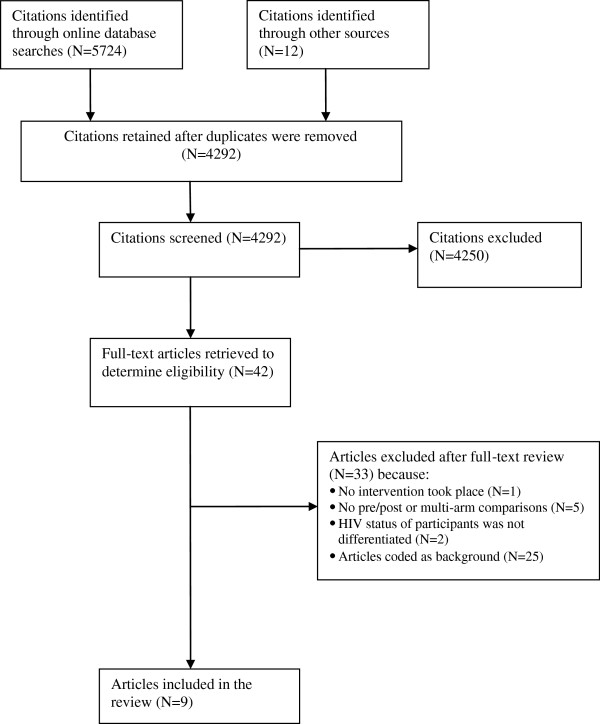
Family planning for HIV positive women: flow chart depicting disposition of study citations (Search Dates: January 1, 1990- January 5, 2012).

An initial search of the literature resulted in 4292 unique citations (Figure 1). These were screened for eligibility and 4250 were removed for failing to meet the inclusion criteria. The remaining 42 articles were determined to merit closer study to determine their eligibility for inclusion in the systematic review. Of these, nine were found to meet all the inclusion criteria and these form the basis of this systematic review. Twenty-five additional articles were included as background articles of importance.

All nine included studies were conducted in Africa: two in Rwanda [24,25], two in Zambia [26,27], two in Kenya [28,29] and one each in Côte d’Ivoire [30], Malawi [31], and Uganda [32] (Table 1). The study designs for the studies included time series analysis [24,30], before/after intervention study designs without a control group [25,26,31], a prospective cohort design [32], a retrospective cohort [29], a non-randomized trial [28] and a controlled trial with group randomization [27]. The mean length of follow-up also varied, from 161 days [25] up to two years [24,28,30,32]. While all studies had pre-post intervention data and all but one followed a cohort of individuals over time, three studies had a control or comparison group, two studies randomly selected participants for assessment and only one randomly assigned groups of participants (couples) to study arms [27].

**Table 1:** Family planning counseling for HIV positive women—final study description

**Study**	**Setting**	**Population characteristics**	**Intervention description**	**Study design**	**Key outcomes reported**
Allen et al., 1993	Rwanda	Pregnant/childbearing women presenting at antenatal or pediatric clinics	HIV testing; 35 minute AIDS educational video, followed by a group discussion. Condoms and spermicide were distributed at no charge. In-depth counseling provided for HIV-infected women, including discussion of family planning measures for those who requested.	Time series study design. Assessments at baseline (N = 1458), 12 months (N = 1254), and 24 months (N = 1352). Stratified random sampling from a consecutive sample of women presenting to prenatal and pediatric clinics.	**Hormonal contraception use**
	Kigali	Age Range: 18-35 years			Baseline: 23%
					12 months: 16% (significance not reported)
Brou et al., 2009	Ivory Coast	Pregnant women presenting for PMTCT	HIV testing. Offered post-test and post-partum family planning during follow up visits, as well as information on sexually transmitted infections (STIs), including HIV/AIDS, and condom use. Postpartum free access to modern contraceptive methods (injectable contraceptives, contraceptive pills, and condoms) from first month.	Time series study design. Assessments at baseline (N = 980) and at 3, 6, 12, 18 and 24 months post-partum Non-random selection of study participants.	**Modern contraception use:**
	Abidjan	Age: Overall Median Age: 26 years (IQR: 22-30 years)			Baseline: 46%
					12 months: 65%
					24 months: 52% (significance not reported)
Chibwesha et al., 2011	Zambia	Women living with HIV on ART	Trained 109 peer counselors to deliver standardized counseling message in context of clinical care, emphasizing dual methods. Referral to separate, on-site family planning department or surgical facility for permanent methods	Before/after study design with no control group. Not clear how long was period from baseline to follow up. Non-random selection of study participants.	**Modern contraception use:**
	Lusaka	Age: Overall Median Age: 34.6 years (IQR: 29.9-39.7)			Baseline: 59%
					Post counseling: 9.8% non-users want contraception; 61.% of these access FP services in 90 days
Hoffman et al., 2008	Malawi	Women living with HIV	Pregnancy testing, VCT, and FP counseling.	Before/after study design with no control group. Participants were followed for 1 year, with assessments at baseline (N = 227), 1 week, and 1, 3, 6, 9 and 12 months after VCT. N = 200 at study completion. Non-random selection of study participants.	**All contraceptive use:**
	Lilongwe	Age: Overall Median Age: 26 years (IQR: 23 to 30 years)			Baseline: 38%
					12 months: 46% ns
					**Pregnancy incidence:**
					Post only: 14.5 per 100 py
Homsy et al., 2009	Uganda	Women living with HIV on ART	Home Based AIDS Care with referral to government family planning clinic for counseling and contraceptive options. Weekly home visits to manage treatment adherence, monitor clinical signs and refer to clinics as needed.	Prospective cohort design study to compare reproductive intentions, identifying risk factors of pregnancy, contraceptive use, incidence of pregnancy among HIV + women on ART. Assessment at baseline (N = 708) and at 3, 6, 9, 12, 15, 18, 21, 24 (N = 656) and 27 months. Non-random selection of study participants.	**All contraceptive use:**
	Tororo and Busia Districts	Median Age: 37 years			18 months: 75.7%
					24 months: 68.6%
King et al., 1995	Rwanda	Women attending pediatric and prenatal clinics	VCT followed by a 15 minute educational video on contraceptive methods and group discussion. Contraceptive pills, injectable progestins, and Norplant provided free of charge to women who chose to enroll in the FP program.	Before/after study design with no control group. Assessments at baseline (N = 502) and every 3 months following the intervention (the mean length of follow-up was 161 day for hormonal contraceptive use and 363 days for incident pregnancy). Non-random selection of study participants.	**Hormonal contraceptive use:**
	Kigali	Age Range: 20-44 years			Baseline: 16%
					Follow up: 24%
					**Pregnancy incidence:**
					Before: 22%
					After: 9% (significant)
Kosgei et al., 2011	Kenya	HIV + women in AIDS care (39% on ART)	FP services integrated into HIV care in one clinical team; nurses experienced in offering FP services relocated to HIV clinical team; on site delivery of all FP methods except surgical	Retrospective cohort study. Mean (SD) of follow up: 342 days (155) for integrated family planning and 361 day (147) for routine care	**Comparing intensified FP to regular care:** new condom use: 16.7% increase, new FP use including condoms: 12.9% increase;
	Eldoret	Age: mean 32.7, SD 7.2			new FP use excluding condoms: 3.8% decrease; (all significant),
					New pregnancies: 0.1% increase (ns)
Ngure et al., 2009	Kenya	Women in HIV serodiscordant relationships	Free contraceptive methods [oral contraceptive pills, injectables, implants, and intrauterine devices (IUDs)], contraceptive appointment cards to avoid lapses in hormonal contraception, couples and individual contraceptive counseling sessions during routine study visits	Non-randomized trial comparing intervention to comparison group, stratifying by HIV serostatus. Assessments at baseline (N = 1429) and 3-months (N not reported). Non-random selection of study participants (cluster sampling).	**Non condom contraception use:**
	Thika (Intervention Site) and Eldoret Nairobi, Kisumu-(Comparison Sites)	Age: Overall ages not reported			Baseline: 31.5% visits
					Follow up: 64.7% visits
					**Pregnancy incidence:**
					Intervention pre: 13.5 per 100 py
					Intervention post: 8.7 per 100 py (sig)
					HIV negative comparison pre: 21.1 per 100 py
					HIV- comparison post: 11.0 per 100 py (sig)
					(*Increased in both groups at other sites without intensive intervention*)
Stephenson et al., 2011	Zambia	Serodiscordant couples and concordant positive couples. cohabiting for at least 12 months	Two different video-based family planning interventions: information on contraceptive methods, with emphasis on IUD and implant; motivational video modeling desirable future planning behaviours, including pregnancy prevention; on-site access to most methods	Group randomized trial to one of four arms: control, methods only, motivation only, motivation plus methods, No time specified for pre and post intervention follow up	**Motivational and method arm** likely to adopt injectables than OCPs: RRR = 1.65 (CI 1.07-3.44)**; methods arm** more likely to adopt injectables than OCPs: RRR = 1.55, CI 1.03-2.34). **Pregnancy outcomes not studied**
	Lusaka	Ages: Men: 18-65, Women 18-45			

The study populations in the nine studies were drawn from pediatric and perinatal clinics at a major urban hospital [24,25], from a PMTCT programme [30], from clinics that provided family planning, sexually transmitted disease services, or VCT [31], from HIV treatment and care clinics [26,29], from a home-based care study [32] and from an HIV prevention trial sites [27,28]. Some of the studies focused exclusively on unique populations, including HIV seropositive women [26,29,30,32], serodiscordant couples (presenting data from HIV-infected women separately in the analysis) [27,28] or women of unknown status who were tested and counselled as part of the study [24,25,31]. Three of the studies offered counselling and follow-up to partners and couples as well [24,25,27,28].

The interventions reported in these studies varied in the access to contraceptives offered, in both the range of contraceptives available as well as the ease of access. In some studies, a variety of modern contraceptive methods, using the WHO definition [33], was provided on site free of charge [25-29]. In others, a reduced range of contraceptives on site was offered [24] or referral to family planning services was made [32]. In two studies, either the issue of access was not addressed [31] or it was not clear where the access to contraceptives was provided [30]. Studies which provided a broader range of contraceptive options seemed to result in increased use of contraception compared to those that offered referral or access to a reduced range of contraceptives, although we were not able to quantify this comparison.

The studies also varied in the nature and content of the intervention offered. In the study by Allen et al. in Rwanda [24], participants were shown a thirty-five minute pretest counselling video about AIDS and modes of transmission, as well as a demonstration of condom and spermicide use; women were counselled about contraception only if they requested it. King et al. [25] presented participants a fifteen minute educational video on contraception, followed by group discussion. Stephenson et al. [27] presented two different thirty-minute video-based family planning interventions. In other studies, however, counselling about contraception was limited and consisted mainly of informing women of the effect of antiretroviral drugs on fertility and sexuality with referral to a nearby family planning clinic [32].

More intensive family planning counselling was also offered in some studies. This ranged from including family planning in the post-test HIV counselling, pre-natal and post-natal visits, as was done in the study reported by Brou et al. [30], to the most intensive, a multi component strategy that included male partners in family planning counselling and extensive staff training. Weekly meetings on family planning for all staff, use of checklists to prompt family planning discussions, discussions of challenges to use with participants individually and in groups, and review of unintended pregnancies to identify ways to improve the intervention were held [28]. This intervention in Kenya, reported by Ngure et al., resulted in a stronger and more durable effect than was seen in the comparison clinics in the same study or in the other less intensive interventions presented in the other studies reviewed here. Other studies followed a similar strategy of deploying the personnel most experienced in family planning in the HIV treatment and care facilities [26,29], though the outcomes observed were generally less than those seen with the more intensive intervention reported in the Ngure study.

All nine studies measured changes in use of contraception, variously defined as “hormonal contraception use”, “modern contraception use” and “non condom contraception use”. In addition, four studies [25,28,29,31] measured changes in pregnancy incidence, presenting comparisons between pre- to post-intervention for seropositive women, between seropositive and seronegative women, or between seropositive women at intervention sites to seropositive women in non-intervention sites.

All nine studies reported changes in contraception use over time. Though significance figures for pre- to post- changes in contraception use by women living with HIV were not always presented, all but two studies [24,32] presented clear evidence of increases in contraception use by women living with HIV, either compared to a baseline measurement, to women who were not infected with HIV, or to women of similar serostatus from different settings with less intense interventions. For example, Brou et al. [30] showed an increase in modern contraception from 46% at baseline to 65% at 12-month follow-up and 52% at 24-month follow-up; Hoffman et al. [31] showed an increase in all contraceptive use from 38% at baseline to 46% at 12-month follow-up; Kosgei et al. [29] found an increase in condom use and new family planning use including condoms; and King et al. [25] showed an increase in hormonal contraceptive use from 16% at baseline to 24% at follow-up. Only one study [24] found a decrease in hormonal contraceptive use from 23% at baseline to 16% at 12 month follow-up, reflecting a shift away from hormonal contraception towards barrier methods and spermicides in women after testing positive for HIV. The study also suggests that lack of access to hormonal contraception may have been a barrier to its use as only condoms and spermicide were provided at the study site [24]. However, the effects of the interventions waned over time: contraception use at 24 months post-intervention was generally lower than at earlier follow up periods in all cases except the Ngure et al. study in Kenya [28].

Four articles presented pregnancy incidence, calculated as a rate per 100 person years (3 studies) or a simple percentage. In two studies, the rate was presented as pre- to post-intervention comparison [25,28]; significant decreases in pregnancy incidence were found in both studies. King et al. [25] found that pregnancy rates declined from 22% before the intervention to 9% after. Ngure et al. [28] found pregnancy incidence decreased from 13.5 per 100 person years before the intervention to 8.7 per 100 person years after the intervention; a comparison with HIV-negative women in this study also showed significantly decreased pregnancy incidence among those women. Two studies presented post intervention pregnancy incidence only [29,31]. In one of these [29], the incidence of pregnancy increased slightly (but not significantly) in the group receiving the intensified intervention. All four studies reported overall pregnancy incidence and did not report specifically on *unintended* pregnancies.

## Discussion

Given the amount of attention that family planning for women living with HIV has generated in the literature [34], it is notable that so little evidence of the effectiveness of the second element of the PMTCT strategy can be found. Only nine articles over the past twenty-one years met the inclusion criteria for this systematic review. Seven of these articles were published in the past four years, after a long period in which the issue apparently received little attention in the research literature. Clearly, increased attention to the integration of reproductive health and HIV prevention services is attracting researchers as well as advocates.

The evidence contained in these articles is difficult to summarize succinctly. The study populations, interventions and definition of and access to contraception all differed across these studies. Furthermore, the research designs and outcomes presented were different as well. Studies also spanned several decades, thus encompassing vastly altered settings in regards to HIV prevention programming and treatment availability. Despite these challenges, it is possible to draw some broad conclusions. First and foremost, the intensity of the intervention matters: women receiving more intensive interventions were more likely to use contraception. Second, interventions needed to be repeated or reinforced over time to avoid a waning of effect. Without these key features, the effectiveness of the interventions offered decays.

Access to free contraceptive supplies was not a routine feature of these studies. Only six of the nine studies specifically mention providing contraception on site and free of charge [25-30]. Notably, these are the more recent studies, perhaps indicating that access to family planning has been recognized as a key issue in increasing family planning use. The range of contraceptive options also varied across these studies. Most recent studies [26-29] included the full range of contraceptive options that would be offered in most modern family planning clinics in the developed world. Providing easy access to a full range of contraceptive choices for free would seem to be important if the goal is truly to help women living with HIV, particularly women with limited means, reduce their chances of an unintended pregnancy. This is supported by evidence from the Contraceptive Choices study in the United States which found that difficulty in obtaining the chosen method contributes to high rates of discontinuation by contraceptive pill, patch and ring users [35]. In similar fashion, this systematic review suggests that those studies which provided more immediate access to a wider range of contraception produced somewhat better results than those that offered referral or access to a reduced range of contraceptives.

Preventing unintended pregnancies is the goal of increasing contraceptive use among women living with HIV. Only four of the nine studies reported pregnancy incidence at all; none reported on unintended pregnancies. Mathematical modelling has long suggested that the effect of preventing unintended pregnancies in women living with HIV can be equal to or greater than the contribution of the provision of antiretroviral drugs to pregnant women living with HIV in preventing HIV in infants [9,36], even with the adoption of more effective antiretrovirals for the prevention of vertical transmission [37]. With renewed commitment to eliminating HIV in infants, a corresponding increase in attention paid to prevention of unintended pregnancies in women living with HIV by the provision of competent family planning services is needed [13,34]. More research to establish the potential of this element when added to national programmes and the operational challenges entailed is clearly needed. Further, to strengthen the evidence base, research that measures the effect of interventions on the prevention of unintended pregnancies, considers the variability of pregnancy incidence over time, and uses experimental or quasi-experimental designs would be beneficial.

Looking at the interventions offered in these studies over time we observed a change in the understanding of what motivates or facilitates contraception use in women living with HIV. Over time, it has become more widely recognized that women living with HIV respond to learning about their infection in different ways [38] and will accordingly plan their future reproduction and make choices about contraception that best suit them [39]. The earlier studies reviewed here offered more basic interventions: providing women with testing and counselling and knowledge of serostatus, coupled with information about HIV transmission and how to avoid it in pregnancy. The idea implicit in these interventions would seem to be that women who know they are living with HIV will decide not to reproduce and take steps to avoid unintended pregnancies. The more recent studies, conducted and published more than a decade later, reflect a more nuanced understanding of fertility intentions and the factors that contribute to the desire for more children. They may also reflect greater appreciation of the possibility to avoid vertical transmission if women chose to reproduce as well as a greater appreciation of the health benefits of reproductive health services in general for women living with HIV. In addition, the increasing availability of antiretroviral therapy for persons living with HIV in the countries studied may have influenced the perspectives of women and care providers over the period in question. Taken together, it should not be surprising that the later studies offered more nuanced interventions and strived to remove potential barriers to accessing contraception and continuing its use, often through integration with HIV treatment and care.

## Conclusions

The right of reproductive choice for women living with HIV has long been established [40,41], though significant gaps remain between the commitment to choice and the delivery of health services [14]. The evidence reviewed here indicates that this right is increasingly being recognized in the services that are being made available to women living with HIV, while simultaneously raising the question if these services are widely available. It also suggests a recognition that as circumstances and desires for more children change over the course of a woman’s life, more comprehensive interventions of longer duration are needed.

Despite increased recognition of the need for greater integration of sexual and reproductive health services and services for HIV prevention, care and treatment [6,42], most of the studies included in this review were based on secondary analyses of data collected for other primary purposes. As the need to integrate services for sexual and reproductive health with services for HIV prevention becomes more empirically supported [43], and especially as the need for greater attention to preventing unintended pregnancies in women living with HIV increases, it will become more important that the provision of integrated services is well understood and that evaluations of their effectiveness are well designed and implemented [44]. Studies of interventions that are informed by the reproductive desires and perspectives of women on living with HIV, the barriers and challenges they face and their need for long term support will be valuable. Studies that measure not only contraception use at one point in time but over time, that address not only pregnancy incidence but the incidence of unintended pregnancy and that can measure or detect the effects of treatment availability and use on women’s perspectives and understandings of mother to child transmission are essential as well. Attempting to reach the goal of elimination of HIV infections in infants will surely benefit from using the full range of interventions available, including an intensified focus on the reduction of unintended pregnancies in women living with HIV. Family planning counselling is but one intervention needed to meet that goal, but it is an important one and it is insufficiently studied and understood.

## Endnote

^a^The interventions recommended by WHO for the prevention of mother to child transmission are: primary prevention of HIV infection among women of childbearing age; preventing unintended pregnancies among women living with HIV; preventing HIV transmission from a woman living with HIV to her infant; and providing appropriate treatment, care and support to mothers living with HIV and their children and families [2].

## Competing interests

The authors declare that they have no competing interests.

## Authors’ contributions

KO led the analysis and drafted the manuscript. KO, CK, and MS conceived of the study, developed the study protocol, and secured funding. VF coordinated the search, screening, and data extraction procedures and developed figures and tables. All authors jointly oversaw implementation of the study protocol, critically reviewed the manuscript for important intellectual content, and approved the final version.

## Authors’ information

KO is a senior scientist in the Department of HIV/AIDS at the World Health Organization. CK is an Assistant Professor in the Department of International Health at the Johns Hopkins Bloomberg School of Public Health. VF is a doctoral student in the Department of International Health at the Johns Hopkins Bloomberg School of Public Health. MS is a Professor in the Department of Psychiatry and Behavioral Sciences at the Medical University of South Carolina.

## Pre-publication history

The pre-publication history for this paper can be accessed here:

http://www.biomedcentral.com/1471-2458/13/935/prepub
